# Peculiarity of the Mechanism of Early Stages of Photo-Oxidative Degradation of Linear Low-Density Polyethylene Films in the Presence of Ferric Stearate

**DOI:** 10.3390/polym15183672

**Published:** 2023-09-06

**Authors:** Zhiming Wang, Zhongwei Wang, Dayong Liu, Qingzhao Wang

**Affiliations:** 1 College of Chemical and Biological Engineering, Shandong University of Science and Technology, Qingdao 266590, China; wang_zhiming@126.com; 2 College of Materials Science and Engineering, Shandong University of Science and Technology, Qingdao 266042, China; wangzhongwei@fusilinchem.com; 3 College of Chemistry and Pharmaceutical Engineering, Huanghuai University, Zhumadian 466300, China

**Keywords:** ferric stearate, mechanism, PE/FeSt_3_ films, photo-oxidative degradation, DFT calculations

## Abstract

Ferric stearate (FeSt_3_) is very efficient in accelerating polyethylene (PE) degradation, but there is a lack of exploration of its role in accelerating the early stages of polyethylene photo-oxidative degradation. This study aimed to investigate the effect of FeSt_3_ on the photo-oxidative degradation of PE films, especially in the early stages of photo-oxidative degradation. The results show that FeSt_3_ not only promotes the oxidative degradation of PE but also contributes significantly to the early behavior of photo-oxidative degradation. Moreover, the results of the density functional theory (DFT) calculations proved that the C-H in the FeSt_3_ ligand was more easily dissociated compared with the PE matrix. The generated H radicals participate in the coupling reaction of the primary alkyl macro radicals leading to the molecular weight reduction, thus significantly increasing the initial rate of molecular weight reduction of PE. Meanwhile, the transfer reaction of the dissociation-generated C-centered radicals induced the PE matrix to produce more secondary alkyl macroradicals, which shortened the time to enter the oxidative degradation stage. This finding reveals the mechanism by which FeSt_3_ promotes the degradation of PE at the early stage of photo-oxidative degradation. It provides guiding significance for the in-depth study of the early degradation behavior in photo-oxidative degradation on polyolefin/FeSt_3_ films.

## 1. Introduction

Polyethylene film, with excellent characteristics such as low cost, high strength and puncture properties, and light weight, has a wide range of applications in disposable products, packaging films, and agricultural land films [1,2]. The widespread use of polyethylene film has brought a great burden to the environment while facilitating our daily lives. It is well known that polyethylene is extremely difficult to degrade under natural conditions, and most polyethylene products have a very short life span, which causes a large amount of polyethylene waste to accumulate in the environment. Studies have shown that polyethylene waste has become a serious threat to freshwater lakes [3,4], groundwater, soil environments, and marine ecosystems. Recycling of polyethylene film products is not economically viable [5,6], and incineration and landfills create new environmental problems [7,8], which are not sustainable ways to dispose of polyethylene waste. The introduction of a certain amount of carbonyl groups in the polyethylene chain and the addition of suitable co-oxidants are two currently effective strategies to increase the degradation rate of polyethylene. Obviously, the addition of a suitable co-oxidant to polyethylene is more valuable for research, since the former affects the mechanical properties of polyethylene [9,10].

The most common co-oxidants are complexes consisting of transition metals with stearic acid or other organic ligands [11,12,13,14,15]. In particular, stearic acid complexes of iron [16,17,18,19], cobalt [20], and manganese [21] can be added to the polyethylene matrix as co-oxidants to greatly accelerate its degradation rate. Studies have shown that iron stearate is a good photo-oxidation aid to initiate the photo-oxidative degradation of polyethylene, while manganese stearate mainly plays a role in the thermo-oxidative degradation of polyethylene [22]. Additionally, natural light can initiate the degradation of polyethylene even at ambient temperatures. Thus, the degradation of polyethylene is mainly manifested as photo-oxidative degradation in natural environments. This is in contrast to thermal oxygen degradation, which is usually studied in accelerated tests at 70 °C or above, but is difficult to achieve in natural environments [23]. Iron stearate is widely used as a cheap, easy to obtain, and effective photo-oxidation additive to study the photo-oxidative degradation of polyethylene. It is not difficult to understand the reasons for its popularity.

**Scheme 1:**
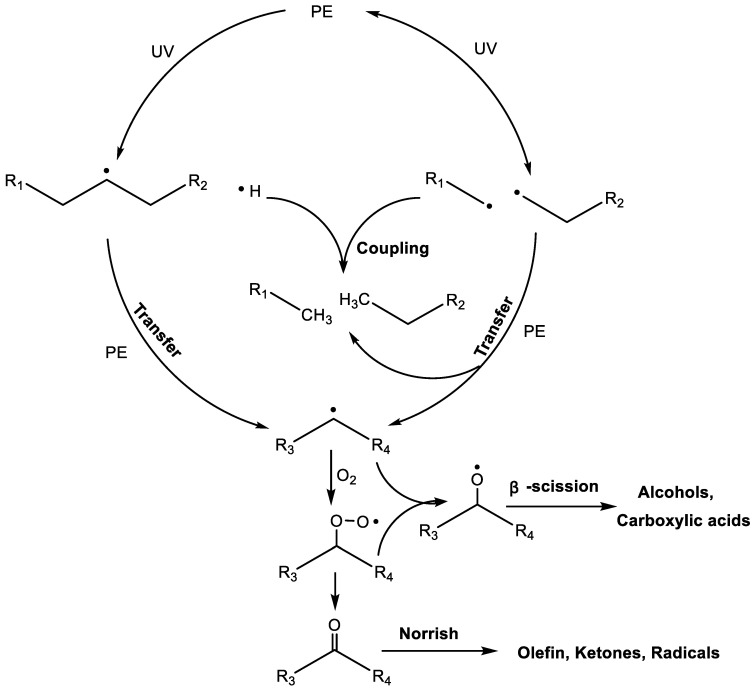
The reaction route involves a decrease in molecular weight in the process of PE pho-to-oxidation degradation. R1, R2, R3, and R4: long-chain alkyl.

The polyethylene photo-oxidative degradation process commences with the cleavage of the C-C and C-H bonds in the molecule [24,25], as shown in Scheme 1. As a result of this process, various reactive radicals, including primary alkyl macroradicals, H radicals, and secondary alkyl macroradicals, are generated. After an early period of accumulation, a large number of secondary alkyl macro-radicals react with oxygen, marking the entry of polyethylene into the oxidative degradation stage. The oxidative phase produces numerous oxygen-containing functional groups such as ketones, aldehydes, carboxylic acids, and esters. At the same time, β-scission of the alkyloxy radicals [26] and Norrish reaction of the carbonyl products result in breakage of the polyethylene backbone, leading to a decrease in molecular weight. In addition, our previous work [27] revealed that the transfer and coupling of primary alkyl radicals originating from photoinitiation also lead to a decrease in the molecular weight of polyethylene.

**Scheme 2:**
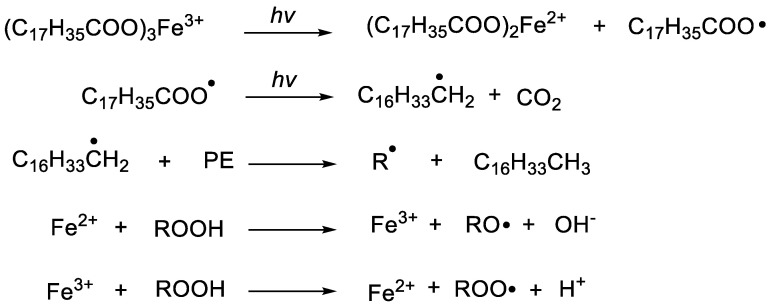
Mechanism of FeSt_3_ in the photo-oxidative degradation of polyethylene.

Previous studies [22,28] have shown that ferric stearate (FeSt_3_) can accelerate the initiation of polyethylene degradation by generating additional alkyl macroradicals. Additionally, it can catalyze the decomposition of alkyl hydroperoxides during the oxidative degradation phase, as illustrated in Scheme 2. However, the current mechanism focuses on the promotion of FeSt_3_ to the oxidative degradation process of polyethylene. There is a lack of studies on the role of FeSt_3_ in the early stages of the photo-oxidative degradation of polyethylene, especially the effect of the rate of molecular weight reduction. In this paper, the photo-oxidative degradation behaviors of PE/FeSt_3_ films were investigated under continuous UV irradiation. The role of FeSt_3_ in the early stage of polyethylene photo-oxidative degradation is investigated in detail, based on which a new mechanism is proposed and justified by density-functional theory (DFT). The findings of this study are a refinement of the photo-oxidative degradation mechanism of FeSt_3_-containing polyethylene, which is a guideline for studying the photo-oxidative degradation behavior of polyethylene-containing transition metal stearate.

## 2. Experimental Sections

Materials: Linear low-density polyethylene (LLDPE) is Sabic’s model 218 W with a molecular weight of 52,231(±158), purchased from Shanghai Hongwei Plastics Co., Ltd., Shanghai, China. Ferric stearate and ethanol were purchased from Qingdao Jingke Instrument Reagent Co., Ltd., Qingdao, China and paraxylene was purchased from Shanghai Macklin Biochemical Co., Ltd., Shanghai, China.

**Scheme 3:**
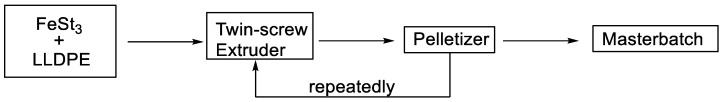
Preparation of LLDPE/Fe-MMT masterbatch.

Preparation of LLDPE/FeSt_3_ (*w*/*w* = 85/15) masterbatch: LLDPE (255.6 g) particles and FeSt_3_ (46.07 g) powder mixture were first extruded using a twin-screw extruder (screw diameter = 19 mm, L/D = 40, Bau Technology, Seoul, Republic of Korea), and the residence time was about 15 min. The operating temperature of the extruder was maintained at 135 °C, 145 °C, 160 °C, 170 °C, and 180 °C from hopper to die, respectively. Cooling and pelletizing: The extrusion was repeated once to obtain 263.5 g of masterbatch containing 15% FeSt_3_. The masterbatch preparation process is shown in Scheme 3.

**Scheme 4:**
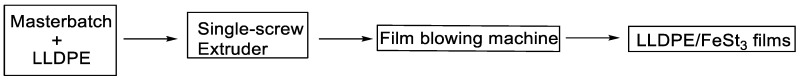
Preparation of LLDPE/Fe-MMT films.

Preparation of LLDPE/FeSt_3_ films: The LLDPE/FeSt_3_ films were blown in a pilot-scale film blow molding facility using a single-screw extruder with a 40 mm screw diameter rotating at 75 rpm. The L/D ratio of the screw was 25:1, and the die length was 100 mm with a 2.5 gap. The operating temperatures in the three zones of the extruder were 160 °C, 170 °C, and 180 °C, respectively, and the die temperature was 190 °C. The thickness of all sample membranes was about 12 μm. Scheme 4 illustrates the preparation process, and the LLDPE films with different FeSt_3_ contents prepared in this study are shown in Table 1.

Photo-oxidative degradation test: The UV irradiation test was carried out in a LUV-2 UV accelerated weathering tester (Shanghai Pushen Chemical Machinery Co., Ltd., Shanghai, China), which was equipped with three UV tubes (Pushen 20 W). The sample was 9 cm from the lamp and the light intensity was 1.65 mW/cm^2^ (measured by CEL-NP2000 optical power meter, Beijing, China). The test temperature was 40 ± 2 °C. The irradiation experiment lasted for 120 h and samples were taken every 24 h for IR and molecular weight tests to monitor the degradation process.

IR test: The IR spectra of the sample films were tested on a Nicolet iS50 FTIR spectrometer equipped with an attenuated total reflection (ATR) accessory. For each sample, an interferogram was obtained using a ZnSe crystal with a reflection angle of 45 to maintain the same probing depth. The measurement range was from 400 to 4000 cm^−1^, with a 4 cm^−1^ resolution and a total of 32 scans.

Determination of the carbonyl index (CI): The carbonyl index (CI) is a parameter used to measure the number of carbonyl compounds formed during the photodegradation of polyethylene film, which can be measured using FT-IR spectroscopy. The IR spectrum of the sample films was obtained using a Nicolet iS50 FT-IR spectrometer coupled with an attenuated total reflectance (ATR) accessory. For each sample, an interferogram was obtained using a ZnSe crystal with a reflection angle of 45 to maintain the same probing depth. The measurement range was from 400 to 4000 cm^−1^, with a 4 cm^−1^ resolution and a total of 32 scans. The carbonyl index (CI) was defined as the absorbance ratio of the carbonyl (approximately 1716 cm^−1^) and methylene groups (1468 cm^−1^) according to the equation [29,30] CI = A1716 cm^−1^/A1468 cm^−1^. The CIs for the LLDPE films were calculated according to the baseline method [31].

Morphological characterization: The morphological structures were characterized by scanning electron spectroscopy/energy dispersive spectroscopy (SEM/EDS, Apreo S HiVac, Thermo Fisher, Waltham, MA, USA).

Molecular weight test: The change in molecular weight was measured according to the viscosity method using an Ubbelohde viscometer (capillary internal meridian: 0.5–0.6 mm). Experimental conditions: Paraxylene (solvent), T = 105 °C, the concentration of PE in paraxylene is about 0.002 g/mL. The viscometric average molecular weight (M_v_) values of the samples were obtained by using the equation [32]: [η] = 0.0165$M_{v}^{0.83}$.

Computational procedure: Calculation commands for all relevant compounds are submitted to Gaussian 16. Geometric optimization of all involved structures was performed at the accuracy level of PBE0/6-311G**. After all geometric optimizations are performed without imposing any geometric constraints, Gaussian 16 software is used to calculate the bond dissociation energy (C-H) of all molecules compounds by using the higher theoretical computational accuracy of PBE0/def2TZVP.

## 3. Results and Discussion

### 3.1. Analysis of Irradiation Test Results

**Figure 1:**
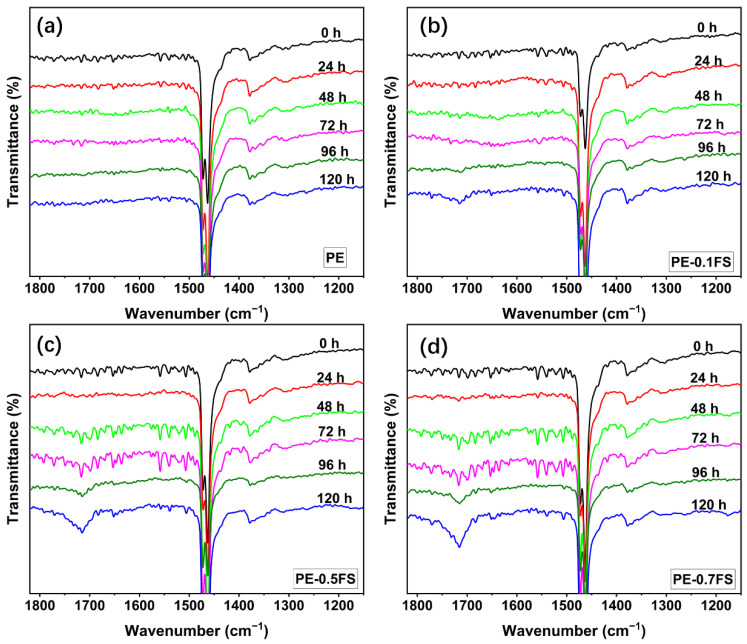
FT-IR spectra under different UV irradiation times. (**a**): LLDPE film; (**b**): PE-0.1FS film; (**c**): PE-0.5FS; (**d**): PE-0.7FS.

PE sample films containing FeSt_3_ at concentrations of 0, 0.1, 0.5, and 0.7% were used in a 120 h UV irradiation experiment and analyzed by FTIR-ATR, and the result is shown in Figure 1. For the pure LLDPE film, weak vibration peaks belonging to carbonyl compounds are not detected until the end of the UV irradiation experiment. It indicates that the pure PE samples passed through a rather long photochemical reaction stage before entering the oxidative degradation stage. In the photoreactive stage, H radicals and alkyl macroradicals are formed by the cleavage of the C-H bond or C-C bond that participates in the subsequent oxidation reaction. With the progress of the experiment, a large number of secondary alkyl macroradicals accumulated in the PE matrix and reacted with oxygen to generate alkyl peroxy macroradicals, and PE entered the auto-oxidative degradation stage. Therefore, in the absence of a prooxidant, it takes a considerable period for polyethylene to initiate oxidative degradation to produce a variety of oxygenated products.

The IR spectra of polyethylene films with 0.1%, 0.5%, and 0.7% additions as a function of UV irradiation time are shown in Figure 1b–d, respectively. It is clear from the figures that new carbonyl product peaks can be detected earlier in the sample containing FeSt_3_ compared to the pure polyethylene film. An obvious new peak appears at 1715 cm^−1^ after 24 h UV irradiation that belongs to the C=O stretching vibration of a ketone group [33] and grows in intensity with extended UV irradiation, which indicates that the oxidative degradation process is underway. As the irradiation time increases other new carbonyl product peaks appear successively at 1733 cm^−1^, 1701 cm^−1^, and 1780 cm^−1^, which are assigned to C=O stretching vibrations in aldehydes, carboxylic acid groups, and lactones, respectively [34,35], Table 2 lists the infrared vibrational peaks involved and their attribution. Moreover, the diversity and area of the vibration peaks in the carbonyl region of polyethylene increased significantly with the increase of FeSt_3_ content. This suggests that the addition of FeSt_3_ to the polyethylene matrix can not only shorten the time for polyethylene to enter the oxidative degradation stage but also accelerate the oxidative degradation rate. This seems to be more in line with the description of the existing mechanism for the accelerated photo-oxidative degradation of polyethylene by FeSt_3_.

**Figure 2:**
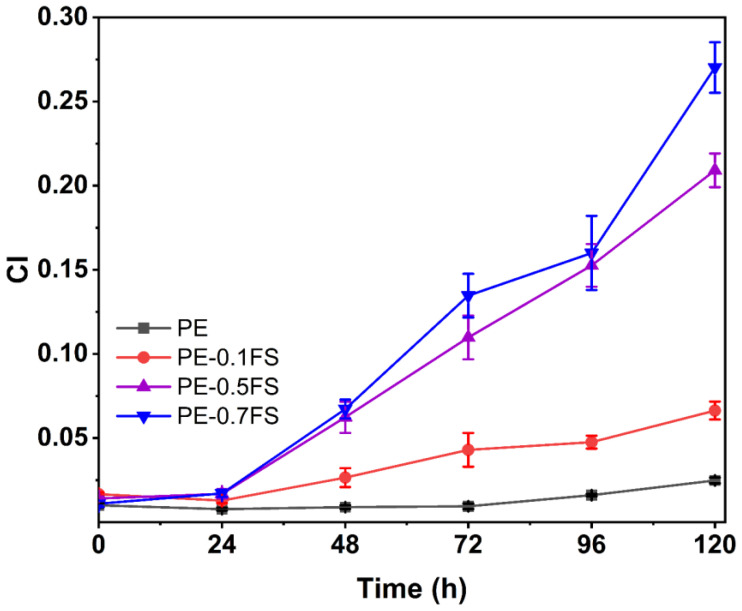
The carbonyl index changes of pure PE film and PE-FS sample films under different UV irradiation times.

The carbonyl index is the most commonly used parameter to detect the degree of polyolefin degradation. For the photo-oxidative degradation of polyethylene, the carbonyl index can visualize the oxidative degradation process of polyethylene. The results of carbonyl index variation for different LLDPE samples are shown in Figure 2. The carbonyl index of the samples showed different degrees of increasing trends with the increase in UV irradiation time, and the carbonyl index of all the samples containing FeSt_3_ was much higher than those of the pure PE samples. In addition, the carbonyl index of pure PE samples started to increase slowly only after 72 h of UV irradiation. In contrast, a significant increase in carbonyl index was observed in all the samples containing iron stearate within 24 h, and the rate of increase was proportional to the concentration of iron stearate. This indicates that even under UV irradiation, pure PE requires a long time to undergo slow oxidative degradation, while the introduction of FeSt_3_ not only greatly shortens the time to enter oxidative degradation but also accelerates the oxidative degradation process. This may be related to the fact that the Fe^3+^/Fe^2+^ redox pair can catalyze the decomposition of alkyl peroxides under UV irradiation [36]. In contrast, the explanation for the shortening of the time for polyethylene to enter the oxidative degradation phase by FeSt_3_ is less clear from the extant mechanism.

### 3.2. Analysis of Molecular Weight Changes

**Scheme 5:**
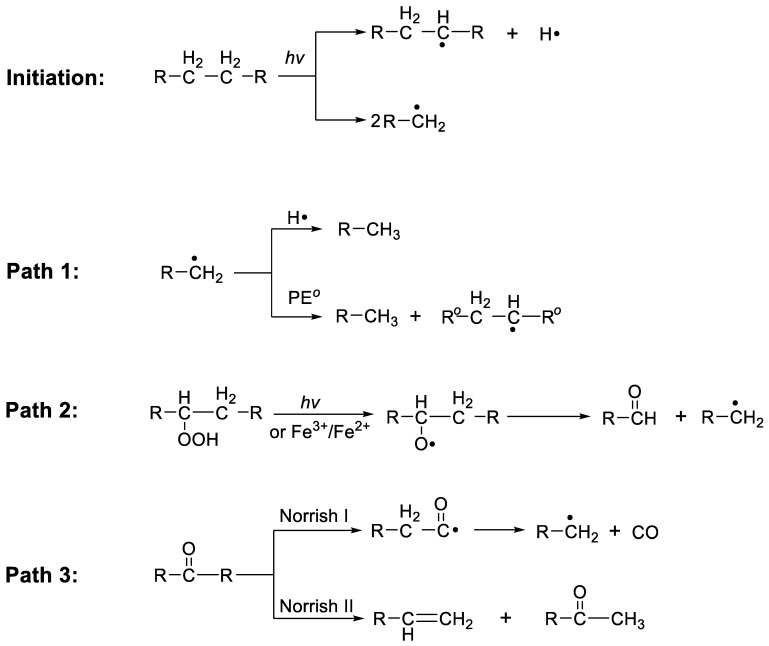
Mechanism of molecular weight reduction of polyethylene during the photo-oxidative degradation process.

The decrease in molecular weight due to the scission of the molecular backbone is an important signal of polymer degradation. Decreased molecular weight is also one of the necessary conditions for polymers to be recognized by microorganisms and metabolically decomposed into carbon dioxide and water. The reactions involving molecular weight reduction during the photo-oxidative degradation of polyethylene are shown in Scheme 5. There is a temporal sequence in the occurrence of these three reactions, which are coupling and transfer of primary radicals in the photoreaction phase (**Path 1**), β-scission of alkoxy radicals in the oxidation phase (**Path 2**), and Norrish reaction of oxidation products (**Path 3**). These reactions resulted in an overall decreasing trend in the molecular weight of polyethylene throughout the photo-oxidative degradation process.

**Figure 3:**
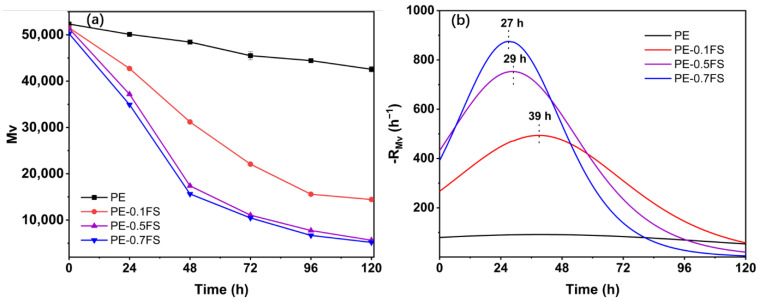
(**a**) Molecular weight changes of LLDPE film and PE-FS sample films under different UV irradiation times and (**b**) the corresponding rate curves.

Figure 3 shows the change curves of molecular weights and the corresponding change rate curves during the photo-oxidative degradation of different samples. The molecular weight values of all samples at different times are listed in Table 3. From Table 3, it can be seen that the initial molecular weights of the FeSt_3_-containing samples did not change much compared to the pure PE. It also reflects that FeSt_3_ is not sensitive to the thermo-oxidative degradation of polyethylene, and some earlier studies [37,38] reported similar results. Therefore, polyethylene containing FeSt_3_ possesses excellent processing stability compared to other transition metal stearates.

As can be seen from Figure 3, the molecular weight of pure LLDPE samples changed very little throughout the photo-oxidative degradation experiments, and the corresponding rate curves show that the molecular weight reduction rate of pure polyethylene remained almost constant. The incorporation of FeSt_3_ is significantly effective in increasing the rate of reduction of molecular weight of polyethylene. As shown in Figure 3b, the initial rate of molecular weight reduction of all FeSt_3_-containing samples is significantly higher than that of pure PE samples, which indicates that FeSt_3_ has an accelerating effect on **Path1** in Scheme 5. After different irradiation times, the rate increased to the maximum value and the higher the FeSt_3_ content the shorter the time required, for contents of 0.1%, 0.5%, and 0.7% corresponding to 39 h, 29 h, and 27 h, respectively. It is because the samples enter into the oxidative degradation stage, and the occurrence of **Path2, Path3** enhances the molecular weight lowering rate of the polyethylene. This indicates that FeSt_3_ plays an important role in increasing the rate of molecular weight reduction in the photo-oxidative degradation of polyethylene, and it is also prominent in the early stage of the photo-oxidative degradation of polyethylene.

### 3.3. Study on the Enhancement of FeSt_3_ in the Initial Photo-Oxidative Degradation of Polyethylene

The above experimental phenomena indicate that FeSt_3_ plays an important role in the various stages of polyethylene photo-oxidative degradation. However, the mechanism of enhancement of polyethylene degradation by FeSt_3_ at the early stage of photo-oxidative degradation is not clear. The photo-oxidative degradation of polyethylene begins with the cleavage of the C-C and C-H bonds in the matrix, and the resulting primary alkyl macro-radicals, secondary alkyl macroradicals, and H radicals directly affect the degradation properties of polyethylene. Primary alkyl macroradicals are generated by UV-induced cleavage of the C-C bond, which is independent of the presence or absence of FeSt_3_. Therefore, we calculated the C-H bond dissociation energies in FeSt_3_ ligands using density-functional theory.

**Scheme 6:**
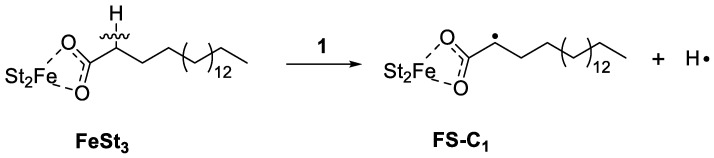
C-H bond cleavage reactions in FeSt_3_ ligands.

As shown in Scheme 6, the C-H dissociation of the carboxylate proximity in the FeSt_3_ ligand generates an H radical and a C-centered radical (FS-C_1_ radical). The structure optimization of the molecules before and after C-H dissociation was first done with a computational accuracy of PBE0/6-311G**, and all the structures successfully converged, and no imaginary frequencies appeared. This indicates the reasonableness of the structure optimization. Subsequently, single-point energy calculations were done for all the optimized structures at the PBE0/def2-TZVP calculation level.

The results based on DFT calculations show that the dissociation energy of the carboxylate-critical C-H bond in the FeSt_3_ ligand is 373 kJ/mol, whereas the dissociation energies of the C-C bond and C-H in the pure polyethylene are 375 kJ/mol and 420 kJ/mol, respectively [39]. It suggests that FeSt_3_ is more prone to give H radicals as compared to polyethylene. In addition, the primary alkyl macroradical pairs dissociated from C-C bonds are more inclined to recombine into C-C bonds in situ due to the limitation of radical mobility in the solid-phase matrix. Therefore, the coupling reaction between primary giant radicals and H radicals in a pure polyethylene matrix mainly depends on their collision probabilities. This is the reason for the low rate of molecular weight reduction of pure polyethylene at the initial stage of photo-oxidative degradation. It is noteworthy that the dissociation energy of C-H in FeSt_3_ ligands is comparable to that of C-C bonds in polyethylene. In this way, the probability of collision of primary alkyl macroradicals generated by polyethylene molecules that are in the vicinity of FeSt_3_ with H radicals is significantly increased. The molecular weight reduction rate curves of different samples in Figure 3b also demonstrated that the initial rate of molecular weight reduction of all samples containing FeSt_3_ is significantly higher than that of pure polyethylene samples.

**Figure 4:**
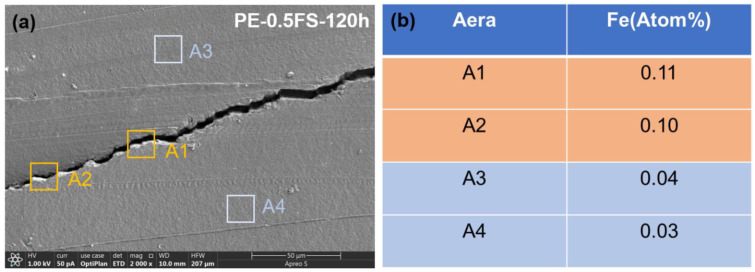
(**a**) Picture of crack on PE-0.5FS after 120 h UV irradiation; (**b**) the relative atomic content of Fe element.

The rapid reduction of molecular weight in polyethylene leads to numerous breaks in the polyethylene backbone and subsequently results in many cracks on the polyethylene film surface. This crack generation was confirmed by scanning analysis of the PE-0.5FS-120 h sample film surface, as shown in Figure 4a. Energy dispersive spectroscopy (EDS) analysis of C, O, and Fe elements was conducted in four random regions, both cracked and non-cracked, yielding the relative atomic content of Fe elements in each region, as depicted in Figure 4b. The results demonstrate that the cracked area exhibits significantly higher Fe content than the uncracked area, implying a faster molecular weight reduction of polyethylene surrounding FeSt_3_. Overall, these findings indicate that FeSt_3_ plays a crucial role in accelerating the molecular weight reduction rate of polyethylene during its early photo-oxidative degradation stages, owing to its ability to generate H radicals more easily.

**Scheme 7:**
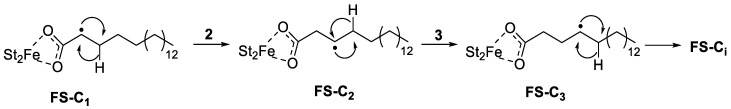
FS-C_1_ radical transfer along the ligand alkyl chain.

**Figure 5:**
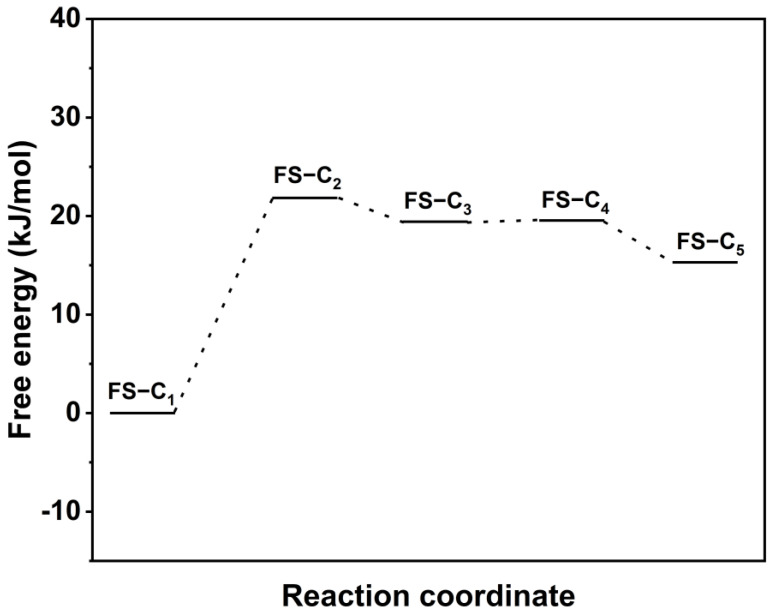
Gibbs free energy diagrams of different FS-C radicals.

The production of H radicals from C-H dissociation in FeSt_3_ ligands contributes to the rate of molecular weight reduction of polyethylene. Therefore, the fate of the FS-C_1_ radicals is also worth investigating. The FS-C_1_ radical can be sequentially transferred along the ligand alkyl chain to generate a new radical (FS-C_i_ radical) as shown in Scheme 7. As can be seen from Figure 5, the Gibbs free energy of the transition from FS-C_1_ radical to FS-C_2_ radical is only 21.8 kJ/mol, and the subsequent transitions are almost all spontaneous processes. It indicates that the FS-C_1_ radical generated by cleavage can rapidly undergo intramolecular radical transfer along the ligand alkyl chain.

**Scheme 8:**
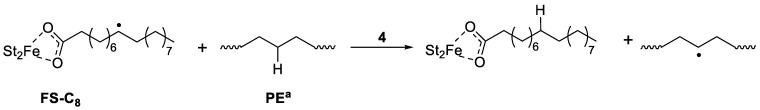
Free radical transfer process between FS-C radical and neighboring polyethylene molecules. PE^a^: Adjacent PE molecular chain segments.

In addition, the long-chain alkanes in the FeSt_3_ ligands are sufficiently interwoven and entangled with the polyethylene matrix molecules during processing. This means that the FS-C radicals can undergo intermolecular radical transfer reactions with neighboring polyethylene molecules. The intermolecular radical transfer process between FS-C_8_ radicals and neighboring polyethylene chain segments (34 C atoms) as shown in Scheme 8 has been theoretically calculated using DFT. The Gibbs free energy of this process is extremely low at −1690 kJ/mol, which means that it can occur spontaneously as long as the H atoms in the polyethylene molecular chain are close enough to the C radicals. Therefore, the radical transfer reaction between FS-C and PE contributes to the generation of more secondary alkyl macro radicals, which are the key radicals for the oxidative degradation of the initiator polyethylene. UV irradiation experiments have also demonstrated that FeSt_3_-containing samples enter into the oxidative degradation stage faster than pure polyethylene samples.

Therefore, the H radicals and FS-C radicals generated by the dissociation of C-H bonds in the ligand are the keys to the role of FeSt_3_ in the initial stage of polyethylene photo-oxidative degradation. The former can significantly increase the rate of molecular weight reduction of polyethylene, and the latter can generate more secondary alkyl macro radicals by intermolecular radical transfer with neighboring polyethylene molecules to shorten the time of entering the oxidation stage and accelerate the whole degradation process.

## 4. Conclusions

In our study, the photo-oxidative degradation behaviors of PE/FeSt_3_ films were investigated under continuous UV irradiation. The rate of photo-oxidative degradation of each PE/FeSt_3_ film is much faster than that of the pure LLDPE film. FeSt_3_ not only enhances the oxidative degradation process, but also contributes significantly to the early stage of polyethylene photo-oxidative degradation as well. Due to the lower C-H bond dissociation energy in the ligand, FeSt_3_ is more likely to give H radicals compared to polyethylene, which can significantly increase the rate of molecular weight reduction of polyethylene. The transfer reaction of FS-C radicals can induce the polyethylene matrix to produce more secondary alkyl macro radicals, which greatly shortens the time for a polyethylene to enter the oxidative degradation stage, thus accelerating the photo-oxidative degradation process of the whole polyethylene. The rationality of the involved C-H dissociation and the transfer process of FS-C radicals was verified by DFT. This finding reveals the mechanism of FeSt_3_-promoted polyethylene degradation in the early stage of photo-oxidative degradation. It is a complement and improvement to the existing mechanism of FeSt_3_-promoted photo-oxidative degradation of polyethylene.

## Figures and Tables

**Table 1:** Compositions of the sample films used in this work.

Sample Name	LLDPE (g)	Masterbatch (g)	FeSt_3_ (wt%)
PE	600	0	0
PE-0.1FS	596	4	0.1
PE-0.5FS	580	20	0.5
PE-0.7FS	572	28	0.7

**Table 2:** Correlated infrared vibrational peaks and their attribution.

Wavenumber (cm^−1^)	Attribution
1780	lactones
1733	aldehydes
1716	ketones
1701	carboxylic acids

**Table 3:** Mv changes of PE and PE/Fe-MMT films under different UV irradiation times.

Sample	Mv at UV Irradiation Time					
	0 h	24 h	48 h	72 h	96 h	120 h
PE	52,336	50,111	48,449	45,502	44,448	42,571
PE-0.1FS	51,533	42,733	31,198	22,067	15,575	14,428
PE-0.5FS	51,376	37,182	17,386	11,054	7739	6674
PE-0.7FS	50,274	34,929	15,626	10,454	5643	5174

## Data Availability

Not applicable.

## References

[1] Singh B., Sharma N. (2008). Mechanistic Implications of Plastic Degradation. Polym. Degrad. Stab..

[2] Peacock A.J. (2000). Handbook of Polyethylene: Structures, Properties and Applications.

[3] Eriksen M., Mason S., Wilson S., Box C., Zellers A., Edwards W., Farley H., Amato S. (2013). Microplastic pollution in the surface waters of the Laurentian Great Lakes. Mar. Pollut. Bull..

[4] Medrano D.E., Thompson R.C., Aldridge D.C. (2015). Microplastics in freshwater systems: A review of the emerging threats, iden-tification of knowledge gaps and prioritisation of research needs. Water Res..

[5] Roy P.K., Hakkarainen M., Varma I.K., Albertsson A.C. (2011). Degradable Polyethylene: Fantasy or Reality. Environ. Sci. Technol..

[6] Hamad K., Kaseem M., Deri F. (2013). Recycling of Waste From Polymer Materials: An Overview of the Recent Works. Polym. Degrad. Stab..

[7] Serrano D.P., Aguado J., Escola J.M. (2012). Developing Advanced Catalysts for the Conversion of PolyolefinicWaste Plastics into Fuels and Chemicals. ACS Catal..

[8] He P., Chen L., Shao L., Zhang H., Lü F. (2019). Municipal solid waste (MSW) landfill: A source of microplastics? -Evidence of microplastics in landfill leachate. Water. Res..

[9] Ramisa X., Cadenatoa A., Salla J.M., Morancho J.M., Vallés A., Contat L., Ribes A. (2004). Thermal degradation of polypropylene/starch-based materials with enhanced biodegradability. Polym. Degrad. Stab..

[10] Abd El-Rehim H.A., Hegazy E.A., Ali A.M., Rabie A.M. (2004). Synergistic effect of combining UV-sunlight–soil burial treatment on the biodegradation rate of LDPE/starch blends. J. Photoch. Photobio. A.

[11] Lin Y.C. (1997). Study of ultraviolet photooxidative degradation of LDPE film containing cerium carboxylate photosensitizer. J. Appl. Polym. Sci..

[12] Roy P.K., Surekha P., Rajagopal C., Choudhary V.J. (2006). Accelerated aging of LDPE films containing cobalt complexes as prooxidants. Polym. Degrad. Stab..

[13] Roy P.K., Surekha P., Rajagopal C., Choudhary V. (2006). Effect of cobalt carboxylates on the photo-oxidative degradation of low-density polyethylene. Part-I. Polym. Degrad. Stab..

[14] Roy P.K., Titus S., Surekha P., Tulsi E., Deshmukh C., Rajagopal C. (2008). Degradation of abiotically aged LDPE films containing pro-oxidant by bacterial consortium. Polym. Degrad. Stab..

[15] Roy P.K., Surekha P., Raman R., Rajagopal C. (2009). Investigating the role of metal oxidation state on the degradation behaviour of LDPE. Polym. Degrad. Stab..

[16] Subramaniam M., Sharma S., Gupta A., Abdullah N. (2018). Enhanced degradation properties of polypropylene integrated with iron and cobalt stearates and its synthetic application. J. Appl. Polym. Sci..

[17] Pablos J.L., Abrusci C., Marín I., López-Marín J., Catalina F., Espí E., Corrales T. (2010). Photodegradation of polyethylenes: Comparative effect of Fe and Ca-stearates as pro-oxidant additives. Polym. Degrad. Stab..

[18] Rajakumar K., Sarasvathy V., Thamaraichelvan A., Chitra R., Vijayakumar C.T. (2010). Effect of Iron Carboxylates on the Photodegradability of Polypropylene. II. Artificial Weathering Studies. J. Appl. Poly. Sci..

[19] Rajakumar K., Sarasvathy V., Thamaraichelvan A., Chitra R., Vijayakumar C.T. (2012). Effect of Iron Carboxylates on the Photodegradability of Polypropylene. I. Natural Weathering Studies. J. Appl. Poly. Sci..

[20] Roy P.K., Surekha P., Rajagopal C., Chatterjee S.N., Choudhary V. (2005). Effect of benzil and cobalt stearate on the aging of low-density polyethylene films. Polym. Degrad. Stab..

[21] Roy P.K., Singh P., Kumar D., Rajagopal C. (2010). Manganese Stearate Initiated Photo-Oxidative and Thermo-Oxidative Degradation of LDPE, LLDPE and Their Blends. J. Appl. Polym. Sci..

[22] Koutny M., Lemaire J., Delort A. (2006). Biodegradation of polyethylene films with prooxidant additives. Chemosphere.

[23] Ammala A., Bateman S., Dean K., Petinakis E., Sangwan P., Wong S., Yuan Q., Yu L., Patrick C., Leong K.H. (2011). An overview of degradable and biodegradable polyolefins. Prog. Polym. Sci..

[24] Costa L., Bracco P. (2015). Mechanisms of Cross-Linking, Oxidative Degradation, and Stabilization of UHMWPE. UHMWPE Biomateials Handbook Ultra High Molecular Weight Polyethylene in Total Joint Replacement and Medical Devices.

[25] Bracco P., Costa L., Luda M.P., Billingham N.A. (2018). Review of Experimental Studies of the Role of Free-Radicals in Polyethylene Oxidation. Polym. Degrad. Stab..

[26] Hinsken H., Moss S., Pauquet J., Zweifel H. (1991). Degradation of Polyolefins during Melt Processing. Polym. Degrad. Stab..

[27] Wang Z.M., Chen H.M., Zhang Y.P., Wang Q.Z. (2023). Study on the Mechanism of Molecular Weight Reduction of Polyethylene Based on Fe-Montmorillonite and Its Potential Application. Polymers.

[28] Abrusci C., Pablos J.L., Marín I., Espí E., Corrales T., Catalina F. (2013). Comparative effect of metal stearates as pro-oxidant additives on bacterial biodegradation of thermal- and photo-degraded low density polyethylene mulching films. Int. Biodeter. Biodegr..

[29] Yeh C.L., Nikolić M.A.L., Gomes B., Gauthier E., Laycock B., Halley P., Bottle S.E., Colwell J.M. (2015). The effect of common agrichemicals on the environmental stability of polyethylene films. Polym. Degrad. Stab..

[30] Maalihan R.D., Pajarito B.B. (2016). Effect of colorant, thickness, and pro-oxidant loading on degradation of low-density polyethylene films during thermal aging. J. Plast. Film Sheeting.

[31] Gulmine J.V., Janissek P.R., Heise H.M., Akcelrud L. (2003). Degradation Profile of Polyethylene After Artificial Accelerated Weathering. Polym. Degrad. Stab..

[32] Brandrup J., Immergut E.H. (1986). Polymer Handbook.

[33] Khabbaz F., Albertsson A.C., Karlsson S. (1999). Chemical and Morphological Changes of Environmentally Degradable Polyethylene Films Exposed to Thermo-Oxidation. Polym. Degrad. Stab..

[34] Setnescu R., Jipa S., Osawa Z. (1998). Chemiluminescence Study on the Oxidation of Several Polyolefins-I. Thermal-Induced Degradation of Additive-Free Polyolefins. Polym. Degrad. Stab..

[35] Valadez-Gonzalez A., Cervantes-Uc J.M., Veleva L. (1999). Mineral Filler Influence on the Photo-Oxidation of High Density Polyethylene: I. Accelerated UV Chamber Exposure Test. Polym. Degrad. Stab..

[36] Liu X., Gao C., Sangwan P., Yu L., Tong Z. (2014). Accelerating the Degradation of Polyolefins Through Additives and Blending. J. Appl. Polym. Sci..

[37] Abrusci C., Pablos J.L., Corrales T., López-Marín J., Marín I., Catalina F. (2011). Biodegradation of photo-degraded mulching films based on polyethylenes and stearates of calcium and iron as pro-oxidant additives. Int. Biodeter. Biodegr..

[38] Muthukumar T., Aravinthan A., Mukesh D. (2010). Effect of environment on the degradation of starch and pro-oxidant blended polyolefins. Polym. Degrad. Stab..

[39] Andrady A.L. (1997). Wavelength Sensitivity in Polymer Photodegradation. Adv. Polym. Sci..

